# Total synthesis of natural products based on hydrogenation of aromatic rings

**DOI:** 10.3762/bjoc.22.4

**Published:** 2026-01-07

**Authors:** Haoxiang Wu, Xiangbing Qi

**Affiliations:** 1 National Institute of Biological Sciences, Beijing. No. 7, Science Park Road, Zhongguancun Life Science Park, Changping District, 102206 Beijing, China

**Keywords:** aromatic rings, dearomatization, hydrogenation, natural products, total synthesis

## Abstract

Arenes and heteroarenes are easily available building blocks in organic chemistry, and saturation the aromatic ring facilitates synthetic chemists to efficiently synthesize natural products with complex three-dimensional structures. Recent advances in catalyst and ligand design have enabled unprecedented progress in the catalytic hydrogenation of (hetero)aromatic systems. Quinoline, isoquinoline, pyridine, and related substrates can now be reduced with high efficiency and stereoselectivity, providing efficient access to saturated and partially saturated architectures vital to synthetic chemistry. Furthermore, catalytic asymmetric aromatic hydrogenation has facilitated the asymmetric total synthesis of complex natural products and pharmaceutical agents. This review highlights recent advances in catalytic (hetero)arene hydrogenation, with a focus on its application in natural product synthesis.

## Introduction

For decades, a principal objective in natural product synthesis has been the development and utilization of efficient methods to access molecular frameworks with defined stereochemical complexity [1]. Natural products, such as taxol [2], strychnine [3–5], and tetrodotoxin [6–8], which contain complex three-dimensional structures, make total synthesis challenging. The retrosynthetic analysis [9], alongside the evolution of methodologies such as “two-phase synthesis” [10], “biomimetic synthesis” [11], or “protecting-group-free synthesis” [12], has progressively streamlined synthetic strategies. The integration of photochemistry [13] and electrochemistry [14] into total synthesis has further extended the realm. Synthesis of complex natural product structures can promote the discovery of new reactions and the generation of new strategies [15]. However, despite careful design, the primary building blocks used in natural product syntheses are often difficult to prepare or scale-up, constrained by subtle chemical reactivity, functional group compatibility, and control of stereoselectivity. Together, these factors continue to shape the pursuit of concise and practical synthetic routes to complex natural products [16–17].

Arenes and heteroarenes have long been readily accessible, as many can be obtained through industrial synthesis or microbial fermentation. The diverse reactivity of aromatic compounds has made them indispensable starting materials in synthetic and medicinal chemistry [18–20]. In recent years, promoted by the rapid development of asymmetric catalysis, a wealth of reactions applicable to aromatic systems – including substitution reactions, transition-metal-coupling reactions, and even dearomatization [21–23] – have been reported, further extending their utility in complex molecule synthesis.

**Scheme 1:**
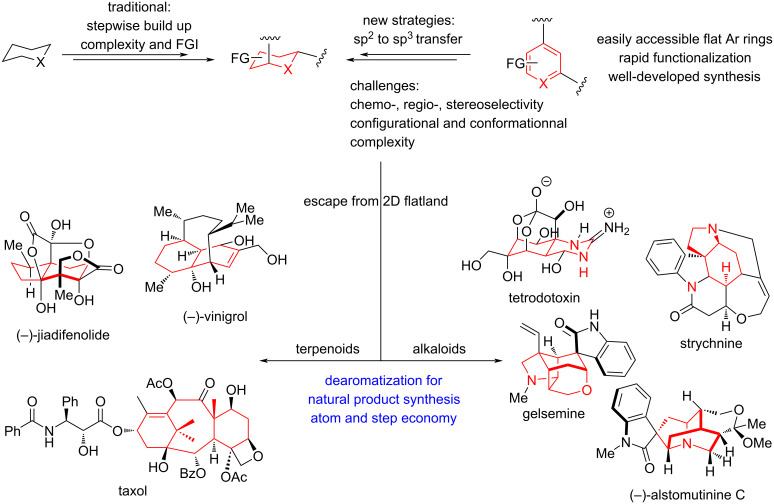
The association between dearomatization and natural product synthesis.

Saturated heterocycles containing sp^3^-hybridized carbons play a pivotal role in natural products as well as pharmaceutical agents, as their three-dimensional structures enable more precise interactions in biological systems [24]. Among the various strategies to access these saturated frameworks, catalytic hydrogenation of unsaturated arenes stands out as the most efficient one: it directly transforms planar sp^2^ systems into three-dimensional sp^3^-rich scaffolds via the shortest possible synthetic route, embodying both step economy and atom economy (Scheme 1) [25].

Although oxidative dearomatization has been a widely studied and powerful approach in synthetic chemistry [21,26–28], researchers have gradually recognized its inherent limitations, including narrow substrate scope, poor selectivity, and low functional group compatibility. Under the circumstance of atom economy and concise synthesis, catalytic hydrogenation of (hetero)arenes has re-emerged as an attractive and practical alternative, offering a complementary pathway to transform planar aromatic systems into saturated or partially saturated, three-dimensional structures.

### Key challenges in hydrogenation of aromatic rings

**Scheme 2:**
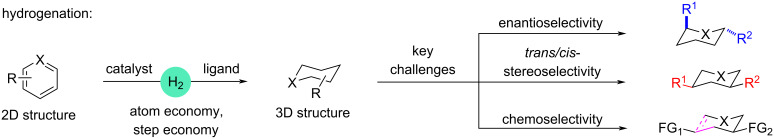
Key challenges in hydrogenation of aromatic rings.

The catalytic hydrogenation of arenes offers a powerful route to disrupt aromaticity and access synthetically valuable intermediates. However, its implementation in strategic synthesis has been hindered by the persistent challenge of controlling selectivity across diverse substrate electronic environments [29]. Reactivity is governed by the electron density of arenes, which directly influences kinetics and product distributions [30–31]. The inability to generically modulate this interaction has confined most catalytic systems to narrow substrate scopes. Addressing this limitation requires the design of catalysts that achieve precise electronic complementarity, enabling selective activation across a broad spectrum of aromatic compounds (Scheme 2) [32].

From the standpoint of scalable synthesis, catalyst cost is a major bottleneck in aromatic hydrogenation. Most state-of-the-art systems depend on expensive transition metals such as platinum, ruthenium, or palladium, inflating the cost of large-scale applications. In addition, precious-metal catalysts often display poor selectivity, while heteroatoms in heteroarenes – acting as Lewis bases – tend to coordinate to the metal center and complicate catalysis. Consequently, designing cost-effective catalytic systems with enhanced efficiency, particularly for the selective hydrogenation of complex substrates, remains an essential direction for future research [33].

Hydrogenation of arenes has rapidly evolved from a specialized transformation into a broadly enabling strategy in complex molecule synthesis, yet a unified perspective connecting recent methodological advances with their strategic applications in the total synthesis of natural products remains lacking. Although several reviews discuss catalyst development for (hetero)arene hydrogenation, they typically treat the topic from a purely methodological angle and seldom address its growing influence on retrosynthetic analysis [30,32,34–36]. Meanwhile, the capability to convert flat, readily accessible aromatic feedstocks into stereochemically defined, three-dimensional scaffolds with exceptional step and atom economy has begun to reshape how to design synthetic routes toward architecturally complex natural products.

The past five years have witnessed significant methodological evolution in the hydrogenation of (hetero)arenes. However, a systematic analysis correlating catalyst innovation with its application in complex natural product synthesis – remains lacking. This review would offer a strategic framework for synthetic chemists and identifies prevailing challenges and future opportunities of the field.

## Review

### The methodologies in hydrogenation of aromatic rings

Encompassing both homogeneous and heterogeneous systems, the hydrogenation of (hetero)arenes has become a cornerstone of modern synthesis for constructing saturated carbocycles and heterocycles [37]. When an appropriate ligand is paired with a transition-metal catalyst, stereoselective hydrogenation of aromatic rings becomes achievable, enabling access to reduced products with well-defined configurations [38]. Although the repertoire of ligands capable of exerting precise stereocontrol remains limited, each provides distinct advantages that suit different metals and reaction conditions. From the perspective of the catalyst itself, a central scientific challenge is how to maintain high catalytic activity throughout the reaction, while preventing deactivation caused by coordination of heteroatoms that may be present in the reduced products.

While the following subsections categorize hydrogenation strategies by substrate class (monocyclic vs fused; heterocycle vs carbocycle), all catalytic systems must still address the fundamental selectivity challenges inherent to arene reduction – chemo-, regio-, and stereoselectivity. Chemoselectivity becomes particularly demanding when reducible groups such as olefins or carbonyls are present, often requiring fine-tuning of the reaction conditions to prevent overreduction. Disruption of aromaticity also creates multiple potential reduction sites, and subtle electronic or substituent differences can lead to regioisomers, especially in polysubstituted or fused systems. In addition, converting a planar sp^2^-hybridized-atoms-enriched framework into three-dimensional sp^3^-hybridized-atoms-enriched architectures inherently generates new stereocenters, making stereocontrol essential in asymmetric variants. As highlighted in the methods below, recent advances in catalyst and ligand design showcase complementary solutions to these selectivity issues, with each substrate class imposing its own opportunities on hydrogenation outcomes.

With these considerations in mind, the following section categorizes catalytic hydrogenation strategies by arene substrate type and highlights representative examples that illustrate recent synthetic advances [35–36].

### Hydrogenation of monocyclic aromatic rings

**Hydrogenation of heterocyclic aromatic rings:** Despite the widespread application of six-membered aromatic heterocycles in various fields of organic chemistry, reports on their catalytic hydrogenation are relatively rare. This is partly due to the high stability of six-membered aromatic heterocycles, such as pyridine, making it difficult to interrupt the aromaticity under mild conditions [34]. Furthermore, the heteroatoms (such as nitrogen and oxygen atoms) within the saturated heterocycles exhibit strong Lewis basicity, potentially complexing and deactivating catalysts. Consequently, the catalytic hydrogenation of six-membered aromatic heterocycles such as pyridine often requires the introduction of substituents or activation of the aromatic heterocycle to facilitate hydrogenation.

**Scheme 3:**
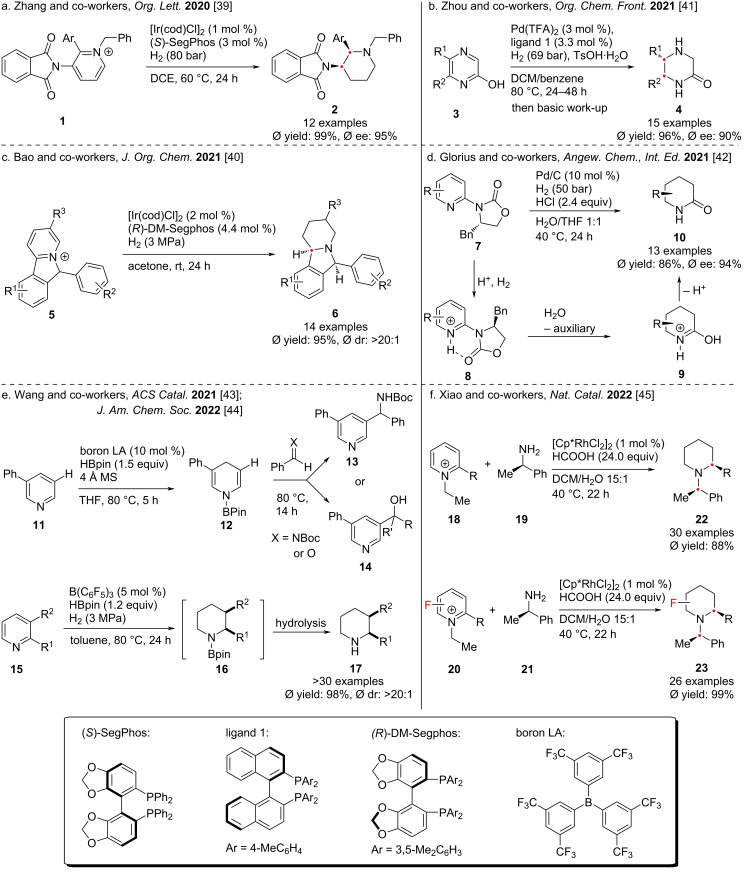
Hydrogenation of heterocyclic aromatic rings.

Between 2020 and 2021, the research groups of Zhang [39], Bao [40], Zhou [41], and Glorius [42] reported four different methods for the reductive hydrogenation of pyridine or pyridine derivatives (Scheme 3). By activating pyridine to a pyridinium salt, the hydrogenated product can be efficiently obtained in the presence of metal catalysts including Pd or Ir and different ligands. In 2021 and 2022, Wang and co-workers reported two impressive hydroboration–hydrogenation reactions catalyzed by FLP (triarylphenylborane) that reduced pyridine compounds **11** or **15** to dihydropyridine compounds **12** or chiral piperidine **17** (Scheme 3) [43–44]. Mechanistic studies demonstrated that the nitrogen atoms present in both pyridine and piperidine complexed with the triarylborane, cleaving the H–H bond and reducing the pyridine. In 2022, Xiao and co-workers reported a study on the conversion of isolated pyridinium salts **18**, **20** to piperidine compounds using [CpRhCl_2_]_2_ as a metal catalyst and formic acid as a hydrogen source (Scheme 3) [45]. Different with other previous studies, this method allows for substituents at the 3-position of pyridine, enabling the rapid preparation of chiral piperidine compounds. It should be noted that in the presence of water, the reaction would undergo transamination with the pyridinium nitrogen moiety while inducing chirality on the ring.

**Scheme 4:**
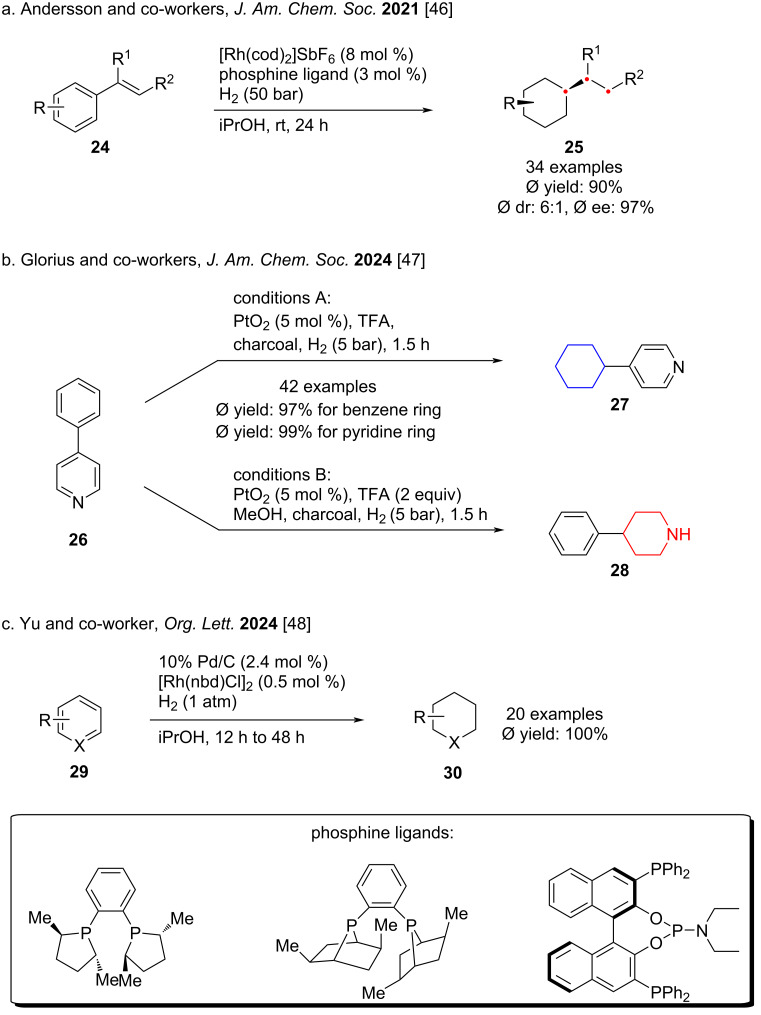
Hydrogenation of the carbocyclic aromatic rings.

**Hydrogenation of carbocyclic aromatic rings:** In 2021, Andersson and co-workers reported a rhodium-catalyst precursor capable of operating in both homogeneous and heterogeneous phases to achieve asymmetric complete hydrogenation of vinyl aromatics – a long-standing challenge in arene reduction (Scheme 4) [46]. By tuning the ratio of phosphine ligand to rhodium precursor, they controlled the formation of distinct catalytic species, which remained mutually compatible, and rationalized facial selectivity through insights from asymmetric styrene hydrogenation. In 2024, Glorius and co-workers developed a chemoselective hydrogenation strategy capable of selectively reducing benzene rings in the presence of pyridine rings (Scheme 4) [47]. Supported by fragment-based screening across a broad substrate set, their method efficiently provides cyclohexane and piperidine frameworks commonly found in bioactive molecules and pharmaceutical intermediates. In the same year, Yu and co-worker described another mild and convenient approach to reduce monocyclic aromatic rings (Scheme 4) [48]. Using [Rh(nbd)Cl]_2_ and Pd/C as catalysts, aromatic hydrocarbons with various functional groups can be hydrogenated at room temperature and 1 atmosphere of hydrogen, thus simplifying the reaction operation and cost.

### Hydrogenation of fused aromatic rings

**Scheme 5:**
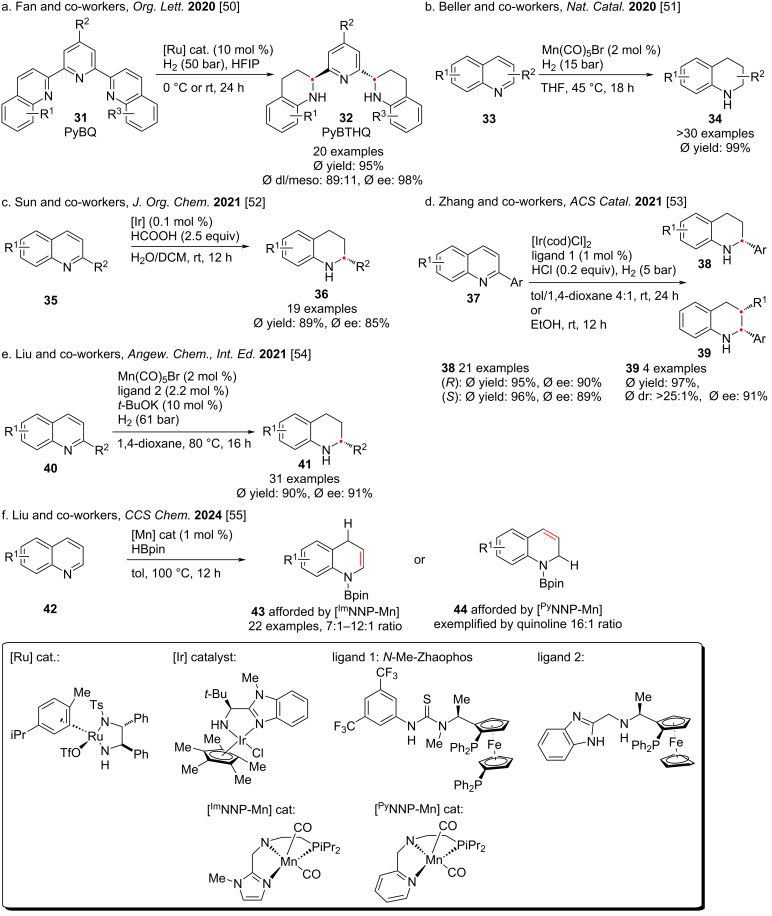
Hydrogenation of the heterocycle part in bicyclic aromatic rings.

**Scheme 6:**
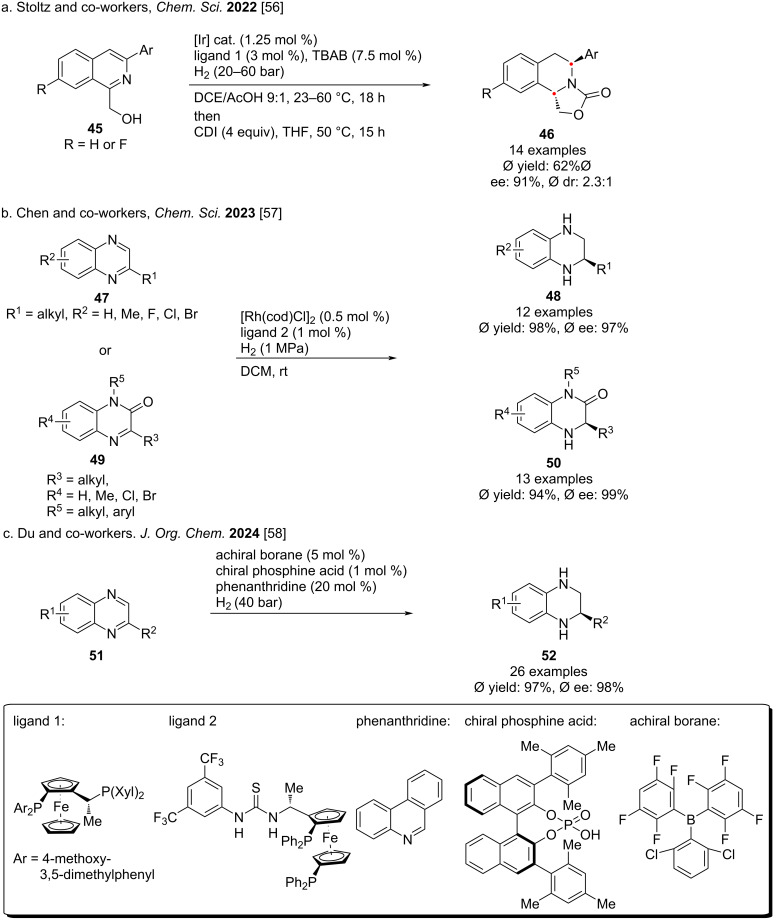
Hydrogenation of the heterocycle part in bicyclic aromatic rings.

**Hydrogenation of the heterocycle part:** Quinoline, one of the most accessible heteroaromatic feedstocks from natural and commercial sources, has long attracted interest in both synthetic and medicinal chemistry. A central challenge, however, is the selective catalytic hydrogenation of quinolines to the synthetically valuable partially or fully saturated derivatives [49]. Over the past years, several research groups worldwide have reported significant progress toward addressing these selectivity and reactivity issues (Scheme 5 and Scheme 6). Nevertheless, despite these advances, the field still suffers from fundamental limitations in terms of substrate scope, stereoselectivity, and scalability, leaving ample room for innovation in catalyst design and mechanistic understanding.

Fan and co-workers have long been dedicated to the asymmetric catalysis of chiral diamine ruthenium complexes. In 2020, they reported the efficient hydrogenation of the polycyclic aromatic compound PyBQ to PyBTHQ using this catalyst in HFIP (Scheme 5) [50]. Under the optimized conditions, only the heteroaromatic part of quinoline was reduced selectively, while the benzene ring and pyridine remained unchanged. In 2020, Beller and co-workers used a manganese catalyst to achieve the hydrogenation of the nitrogen heterocyclic moiety in quinoline, that yielded the target product in near-quantitative amounts [51]. In 2021, the research groups of Sun [52] and Zhang [53] reported the use of iridium or manganese as catalysts to convert quinoline derivatives into tetrahydroquinolines by using dihydrogen or formic acid as the hydrogen source. In 2021 and 2024, Liu and co-workers demonstrated that with [NNP-Mn] catalysts, the hydrogenation of quinoline and its derivatives can proceed with high regio- and stereoselectivity [54–55] (Scheme 5).

In 2022, Stoltz and co-workers reported for the first time the reduction of 1,3-disubstituted isoquinoline compounds **45** to *trans*-quinoxalines **46** (Scheme 6) [56]. By using iridium catalysts and commercially available chiral JosiPhos ligands, a batch of enantioriched *trans*-tetrahydroisoquinolines could be prepared efficiently and stereoselectively. In 2023, Chen in collaboration with Zhang, reported a simple and efficient rhodium–thiourea-catalyzed asymmetric hydrogenation reaction for the synthesis of highly optically pure tetrahydroquinoxaline **48** and dihydroquinoxalinones **50** (Scheme 6) [57]. Due to the mild conditions and broad substrate range, the reaction was scaled up to the gram scale with high yield and high enantioselectivity. Recently, Du and co-workers reported a transition-metal-free asymmetric transfer hydrogenation reaction (Scheme 6) [58]. Under a hydrogen atmosphere, using chiral phosphoric acid and achiral borane as catalysts, they synthesized 2-substituted quinoxalines **52** in high yield.

**Scheme 7:**
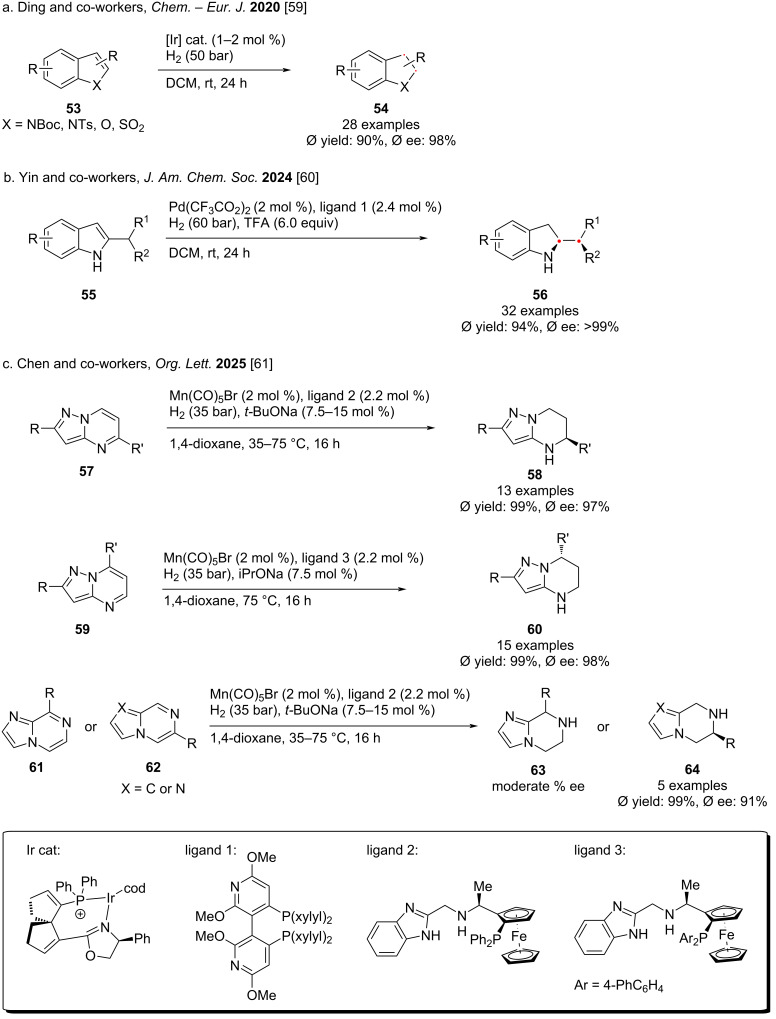
Hydrogenation of benzofuran, indole, and their analogues.

For benzofuran, indole and other heteroarenes, hydrogenation of these easily accessible building blocks is rare. In 2020, Ding and co-workers demonstrated the selective reduction of benzoannelated five-membered heteroaromatic compounds **53** while retaining the benzene ring structure using an iridium catalyst to selectively hydrogenate indoles and benzofurans (Scheme 7) [59]. The hydrogenated products were obtained in 90% yield with 98% ee on average. In 2024, Yin and co-workers reported an innovative palladium-catalyzed asymmetric hydrogenation reaction (Scheme 7) [60]. Using an acid-assisted dynamic kinetic resolution method, they obtained a series of chiral indolines **56** containing exocyclic stereocenters in high yields and excellent enantioselectivity. Mechanistic studies of the reaction revealed that the dynamic kinetic resolution process relies on the rapid interconversion of the enantiomers in the racemic substrate, which in turn relies on the acid-promoted isomerization between the aromatic indole and the nonaromatic exocyclic enamine intermediate. Very recently, Chen and co-workers reported that using a Mn catalyst with different PNN-ligands, multi-nitrogen heteroaromatic compounds, including substituted pyrazolo[1,5-*a*]pyrimidines, pyrrolo[1,2-*a*]pyrazines, and imidazo[1,2-*a*]pyrazines can be hydrogenated efficiently, providing the corresponding reduced products with high enantioselectivity, reactivity, and broad substrate scope [61].

**Hydrogenation of the carbocycle part:** The hydrogenation of the benzene ring in bicyclic aromatic systems has always been very challenging, since the aromaticity of the benzene ring is stronger than that of heteroaromatic rings, and the lack of heteroatoms in the ring makes it even more difficult for the substrate to coordinate with the metal catalyst.

**Scheme 8:**
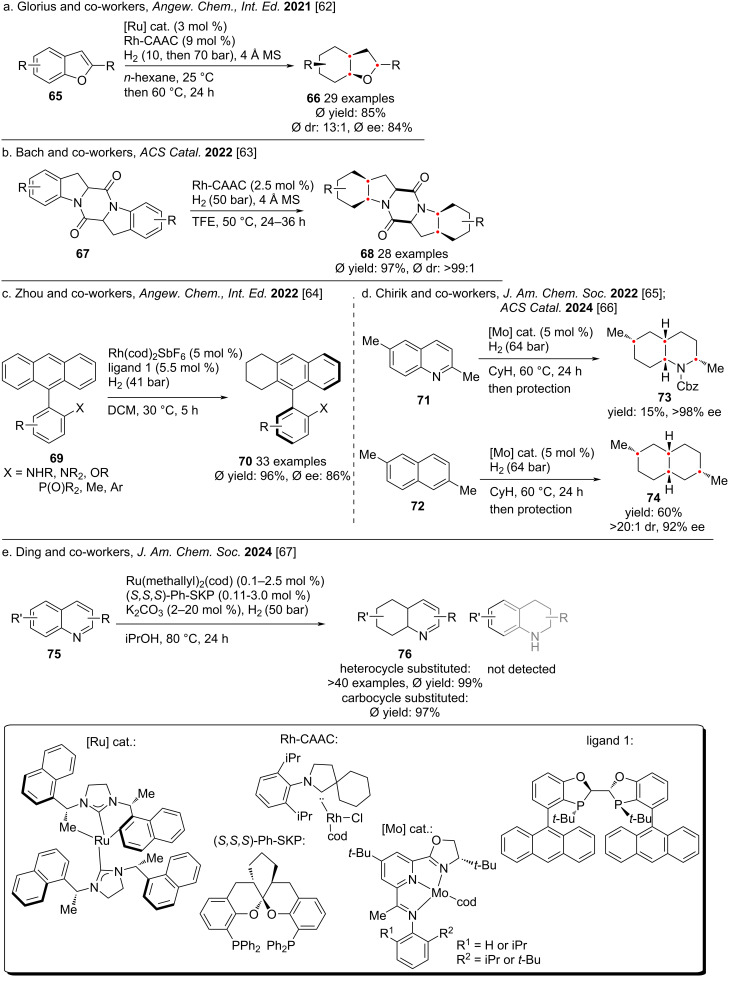
Hydrogenation of benzofuran, indole, and their analogues.

In 2021, Glorius and co-workers reported an enantio- and diastereoselective complete hydrogenation of substituted benzofurans **65** in a one-pot cascade reaction (Scheme 8) [62]. This method facilitates the controlled installation of up to six new stereocenters, producing architecturally complex 6–5 fused ring systems. The key points lie in the utility of a chiral homogeneous ruthenium-*N*-heterocyclic carbene complex, as well as an in-situ activated rhodium catalyst. In 2022, Bach and co-workers reported a modular approach for the highly diastereoselective hydrogenation of symmetrical 2,5-DKP (2,5-dipiperazinone) **67** using a rhodium complex (Scheme 8) [63]. The saturated pentacyclic compound **68** was obtained in high yield and excellent diastereoselectivity. The hydrogen atoms in the final product were all arranged in *cis* configuration. Key features of the reaction include high functional group tolerance and excellent stereocontrol. In the same year, Zhou and co-workers reported a rhodium/bisphosphine-catalyzed asymmetric hydrogenation reaction of all-carbon aromatic rings **69** (Scheme 8) [64]. Through desymmetrization or kinetic resolution, a series of axially chiral cyclic compounds with high enantioselectivity could be synthesized. In addition, the authors also obtained central chiral cyclic compounds by asymmetric hydrogenation of phenanthrene with directing groups.

In 2022 and 2024, Chirik and co-workers reported the use of molybdenum as a metal catalyst to completely hydrogenate substituted naphthalene to saturated 6–6 fused bicycle (Scheme 8) [65–66]. The target decahydronaphthalene was obtained in high yield and high enantioselectivity. In 2024, Ding and co-workers reported a method for the selective hydrogenation of the carbon ring in quinoline to generate hexahydroquinoline using a Ru catalyst (Scheme 8) [67]. When (*S,S,S*)-DKP was used as a ligand, the hydrogenation of the carbon ring was highly selective.

### Reduction of aromatic rings via hydride or electron transfer pathways

Beyond catalytic hydrogenation, aromatic rings can also be reduced through hydride-based or electron-transfer pathways, which provide complementary modes of reactivity that are often orthogonal to metal-catalyzed hydrogenation systems. Classical dissolving-metal reductions such as the Birch reduction convert arenes into 1,4-dihydro intermediates via sequential electron transfer and protonation, enabling regioselective partial dearomatization that is difficult to achieve under hydrogenation conditions. Likewise, hydride reagents – including NaBH_3_CN, DIBAL-H, and other aluminum or borohydride derivatives – have been widely employed for the selective reduction of activated aromatic cations (e.g., pyridinium, quinolinium, or benzopyrylium salts), offering mild and chemoselective access to partially or fully saturated heterocycles. More recent advances in single-electron transfer (SET) chemistry, particularly those mediated by photoredox catalysts or electrochemical systems, have expanded the toolbox further by enabling reductive dearomatization under exceptionally mild conditions and with high functional-group compatibility. Collectively, these hydride- and electron-transfer approaches enrich the landscape of arene reduction by providing tunable control over regioselectivity, degree of saturation, and stereochemical induction – features that are increasingly leveraged in total synthesis to access complex, three-dimensional natural products [68–70].

### Total synthesis based on hydrogenation of aromatic rings

Beyond catalyst and substrate diversity, arene hydrogenation fundamentally proceeds through either partial or complete loss of aromaticity. Partial hydrogenation delivers dihydro- or tetrahydro intermediates that retain useful unsaturation for further transformation. Complete hydrogenation typically requires stronger reductive conditions or more reactive catalyst–ligand systems to overcome higher aromatic stabilization energies. Thus, chemoselectively distinguishing between partial and complete reduction is crucial: depending on the substrate, the catalyst must either halt the process at a defined stage or drive it to full saturation without affecting other functionalities. The following sections highlight representative examples of both modes in complex molecule synthesis.

#### Total synthesis of (±)-keramaphidin B by Baldwin, 1996

**Scheme 9:**
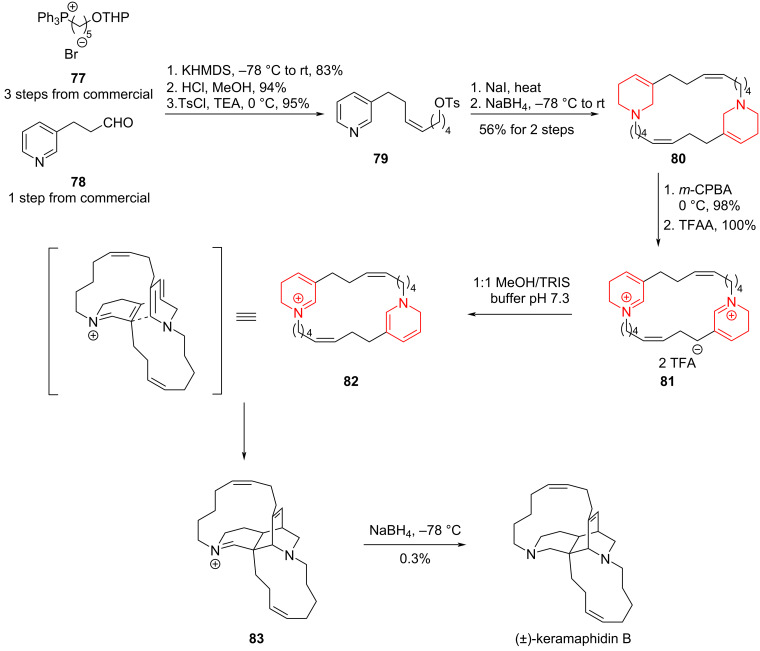
Total synthesis of (±)-keramaphidin B by Baldwin and co-workers.

Although the methodologies for aromatic ring hydrogenations have only recently flourished, the reduction of aromatic rings to obtain saturated aliphatic or heterocyclic rings and their subsequent application in the total synthesis of natural products has a long history. A brilliant early work is Baldwin's total synthesis of the macrocyclic diamine natural product (±)-keramaphidin B in the year of 1996 (Scheme 9) [71–72].

Starting from compounds **77** and **78**, Baldwin and co-workers converted them into pyridine derivative **79** in high yield over three steps including a Wittig reaction and tosylation. Subsequent reduction with sodium borohydride furnished the dimer **80** bearing partially reduced pyridine rings. From **80**, intermediate **81** was prepared via a Polonovski–Polish reaction and isomerization, which, when adopting the proper conformation, spontaneously underwent an intramolecular [4 + 2] cycloaddition to construct the unsaturated bridged ring of (±)-keramaphidin B in a single transformation. Subsequently, the iminium ion **83** was reduced, completing the total synthesis of (±)-keramaphidin B. Although the yield of the [4 + 2] cycloaddition step is not ideal, this work on (±)-keramaphidin B exemplifies the application of an aromatic ring hydrogenation strategy in the total synthesis of natural products and its promising development potential.

#### Total synthesis of ergolines by Vollhardt (1994), Boger (2015), and Smith (2023)

**Scheme 10:**
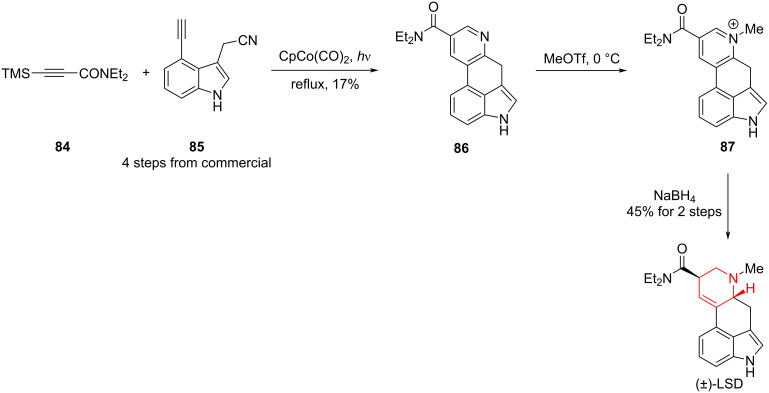
Total synthesis of (±)-LSD by Vollhardt and co-workers.

Dearomative ionic hydrogenations have been widely applied in alkaloid synthesis, including that of the ergot alkaloids – a bioactive compounds family with a long synthetic history [73]. In the synthesis of (±)-LSD by Vollhardt and co-workers, the pyridine part was constructed by a Co-catalyzed [2 + 2 + 2] cycloaddition between alkyne **84** and nitrile **85**, constructing the ergoline core **86**. Methylation with MeOTf provided pyridinium **87**, and subsequent NaBH_4_-mediated hydrogenation selectively generated the tetrahydropyridine and completed the synthesis of (±)-LSD (Scheme 10) [74–75].

**Scheme 11:**
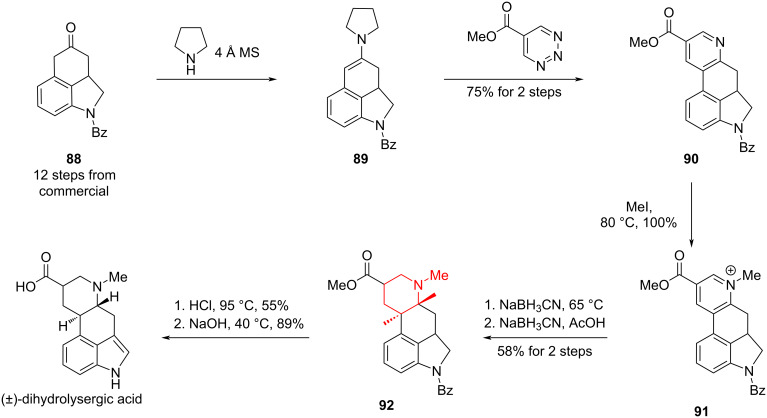
Total synthesis of (±)-dihydrolysergic acid by Boger and co-workers.

In the synthesis of (±)-dihydrolysergic acid, Boger and co-workers constructed the tetracyclic core via an inverse-electron demand Diels–Alder cycloaddition between an enamine derived from ketone **88** and triazine, giving pyridine **90** in 75% yield over two steps [76]. Alkylation with MeI furnished pyridinium **91**, which was hydrogenated in two steps with NaBH_3_CN to the fully saturated piperidine **92**. Acidic removal of the benzoyl group triggered auto-oxidation to the indole, and subsequent hydrolysis of methyl ester delivered the target (±)-dihydrolysergic acid (Scheme 11).

**Scheme 12:**
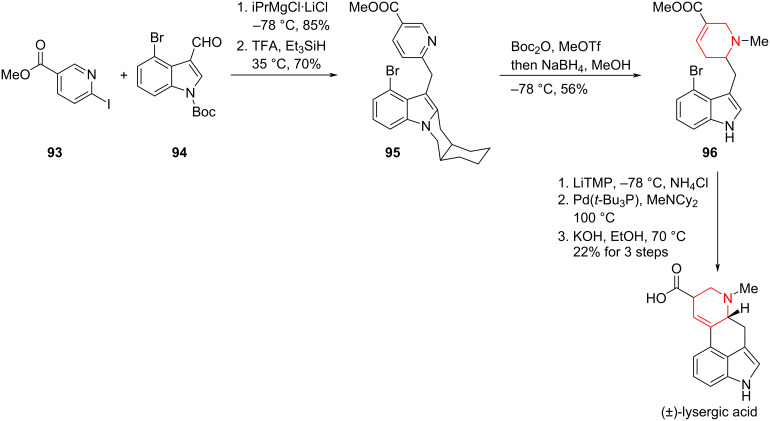
Total synthesis of (±)-lysergic acid by Smith and co-workers.

Smith and co-workers recently reported a six-step synthesis of (±)-lysergic acid [77]. In contrast to previous approaches, the pyridine was introduced via a magnesium–halogen exchange of pyridyl iodide **93**, followed by addition to aldehyde **94**. Subsequent reduction of the resulting benzylic alcohol with TFA/Et₃SiH afforded pyridine **95**, which underwent a one-pot sequence of indole protection, methylation, and hydrogenation to furnish tetrahydropyridine **96**. In contrast to Vollhardt’s synthesis, the unsaturation was misaligned with that of the natural product, most likely arising from protonation during reduction or an isomerization event. Kinetic translocation of the double bond to the correct position enabled an intramolecular Heck reaction, a transformation originally developed by Fukuyama (Scheme 12) [78].

#### Hydrogenation of (−)-tabersonine to (−)-decahydrotabersonine by Catherine Dacquet, 1997

**Scheme 13:**
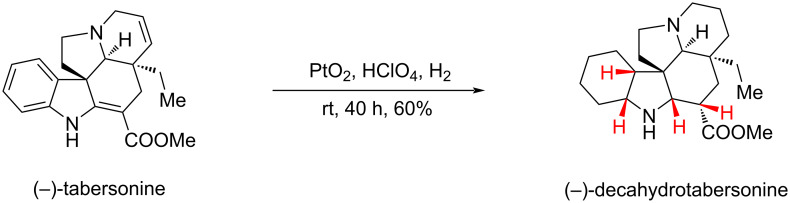
Hydrogenation of (−)-tabersonine to (−)-decahydrotabersonine by Catherine Dacquet and co-workers.

An unavoidable challenge for aromatic ring hydrogenations is controlling the stereochemistry of the product during the hydrogenation reduction process. In 1997, Dacquet and co-workers conducted a hydrogenation reduction of the natural product (−)-tabersonine using platinum dioxide as a catalyst in the presence of perchloric acid to obtain (−)-decahydrotabersonine, a product with a completely reduced aromatic ring (Scheme 13) [79]. Through the structural characterization of the product, the authors found that the hydrogenation was highly stereoselective, yielding only *cis*-hydrogenated products. This suggests that substrate induction during hydrogenation could effectively direct the process, enabling stereoselective product formation.

#### Total synthesis of (±)-nominine by Natsume, 2004

**Scheme 14:**
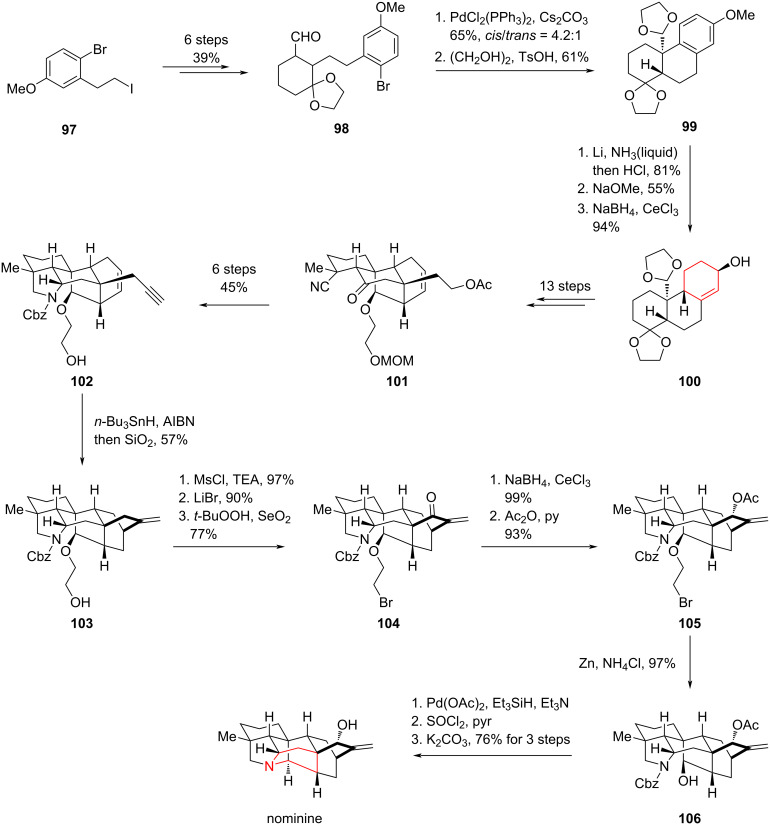
Total synthesis of (±)-nominine by Natsume and co-workers.

Natural products with complex ring systems, such as bridged rings, spirocycles or highly rigid ring systems, have long captivated synthetic chemists. Designing and completing the total synthesis of these molecules not only leads to the development and application of novel methodologies but also elevates the field to a new level, embodying the artistry of synthesis [80]. In 2004, Natsume and co-workers achieved the first total synthesis of the hepta-ring-containing natural product (±)-nominine applying a palladium-catalyzed intramolecular α-acylation and Birch reduction as key steps (Scheme 14) [81–82].

Starting with a simple trisubstituted benzene **97**, they obtained the ketal **98** in 39% yield over six steps. The authors then utilized their own palladium-catalyzed intramolecular α-acylation followed by protection of the carbonyl group to obtain the diketal **99**. Birch reduction of **99** afforded the enone, which was then subjected to a Luche reduction to get the allylic alcohol **100**. A Johnson–Claisen rearrangement and Lewis acid-promoted acetal–ene reaction provided the tetracyclic skeleton **101**. With **101** in hands, a 6 step transformation afforded the alkyne **102** in an overall yield of 45%. After obtaining **102**, the authors used a free radical reaction to initiate a one-step *6-exo-trig* cyclization, constructing the [2.2.2]-bridged ring within the (+)-nominine followed by protection of the primary alcohol as mesylate. Using lithium bromide as the bromine source, an S_N_2 reaction was performed, and the allylic position was oxidized with *tert*-butyl peroxide and selenium dioxide to generate the enone **104**. After obtaining **104**, the unsaturated ketone was stereoselectively reduced to a specifically oriented hydroxy group using a Luche reduction, which was then protected with acetic anhydride to yield **105**. Compound **105** then gave rise to the tertiary alcohol **106** in the presence of zinc powder and ammonium chloride. Finally, through a sequence of removal of the Cbz protecting group, alcohol chlorination, proximal nucleophilic substitution, and deprotection of the secondary alcohol, the first total synthesis of nominine was completed. Although the synthetic route seemed to be lengthy, this challenging total synthesis provided a promising strategy for the subsequent synthesis of this family of natural products.

#### Total synthesis of (+)-nominine by Gin, 2006

**Scheme 15:**
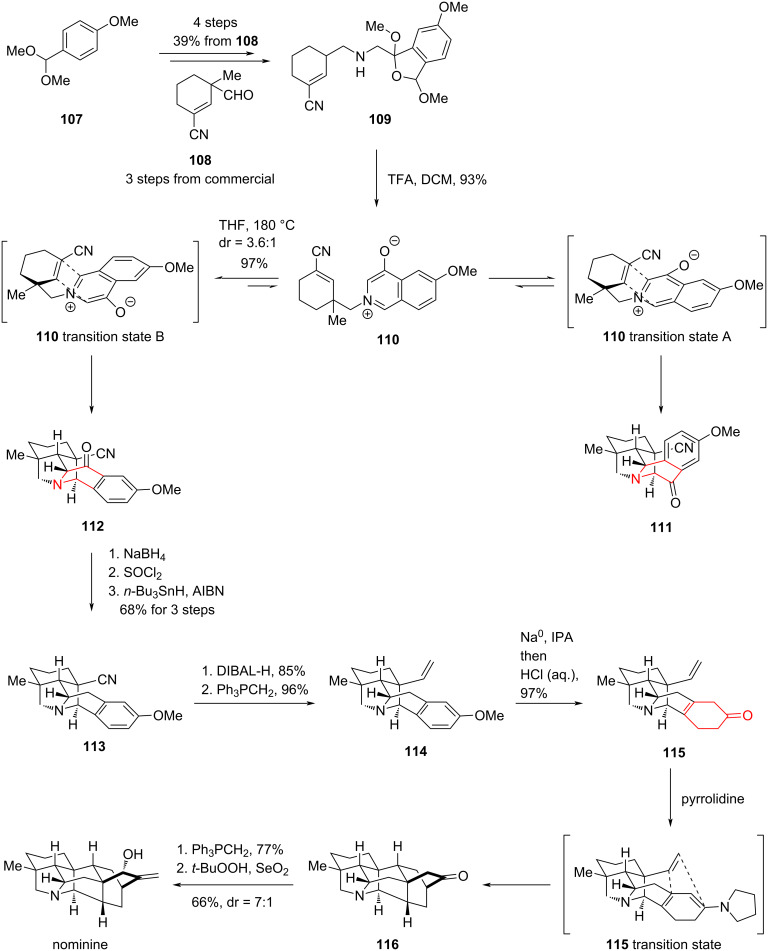
Total synthesis of (+)-nominine by Gin and co-workers.

Only two years after Natsume completed the total synthesis of (+)-nominine, Gin and co-workers reported another total synthesis of (+)-nominine via a brilliant dearomatization strategy (Scheme 15) [83]. Starting from a *para*-disubstituted benzene **107** and an unsaturated aldehyde **108**, they obtained **109** via a Staudinger aza-Wittig reaction. Compound **109** isomerized to the internal salt **110** in the presence of TFA, and **110** spontaneously underwent two different transition states to give the intramolecular [5 + 2] products **111** and **112**, respectively. While the desired cycloadduct **112** was formed as the minor constituent (**112**/**111** = 1:3.6), the isomeric ratio was verified to be the result of thermodynamic selection. The cycloaddition process is reversible under the conditions, thereby permitting iterative thermal re-equilibration of the undesired cycloadduct **111**, which enhanced the formation of **112** while minimizing material loss.

Cycloadduct **112** was converted to **113** in high yield through a three-step sequence of carbonyl hydroboration, alcohol chlorination with thionyl chloride, and radical reduction. Subsequently, reduction of the cyanide group with DIBAL-H and a Wittig reaction afford alkene **114** in 82% yield. Sodium metal was then used as a single-electron reducing agent to provide the second cycloaddition precursor **115**, which was converted to the enamine. The enamine spontaneously underwent an intramolecular cycloaddition to yield the highly rigid [2.2.2]-bridged ring skeleton in ketone **116**. Finally, nominine was generated via a two-step sequence comprising a Wittig reaction followed by *tert*-butyl peroxide/selenium dioxide-mediated allylic oxidation, thereby completing another impressive total synthesis. Relative to Natsume’s strategy, Gin and co-workers leveraged two intramolecular cycloaddition reactions to efficiently construct the carbon framework, resulting in a efficient total synthesis of nominine.

#### Total synthesis of (±)-lemonomycinone and (±)-renieramycin by Magnus, 2005

**Scheme 16:**
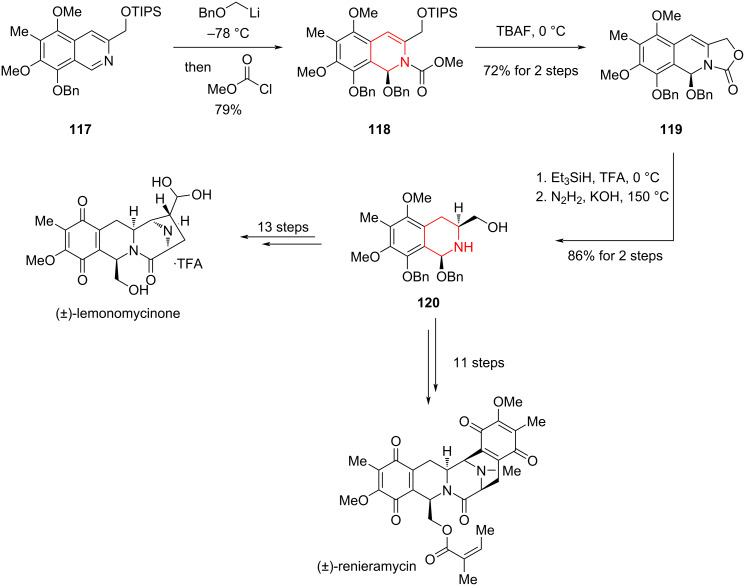
Total synthesis of (±)-lemonomycinone and (±)-renieramycin by Magnus.

Tetrahydroisoquinoline alkaloids have long captivated chemical and biological interest; among them, lemonomycin and renieramycins (A–S) are especially notable for their potent antitumor and antimicrobial activities [84]. In 2005, Magnus and co-worker achieved the total synthesis of two bis-tetrahydroisoquinoline natural products, (±)-lemonomycinone and (±)-renieramycin, featuring as key steps a dearomative nucleophilic addition and diastereoselective hydrogenation of a dihydroisoquinoline (Scheme 16) [85].

Starting from pentasubstituted isoquinoline **117**, the authors utilized a dearomative nucleophilic addition to indirectly reduce the heteroaromatic ring of the isoquinoline to yield compound **118**. This was followed by desilylation with TBAF and spontaneous intramolecular cyclization to yield the tricyclic **119**. With **119** in hand, the double bond was diastereoselectively hydrogenated using Et_3_SiH and TFA, followed by hydrazinolysis to generate alcohol **120**. Starting from the common intermediate **120**, the total syntheses of renieramycin and lemonomycinone were accomplished through 11 and 13 steps, respectively.

#### Total synthesis to the alkaloids GB13 by Sarpong, 2009

**Scheme 17:**
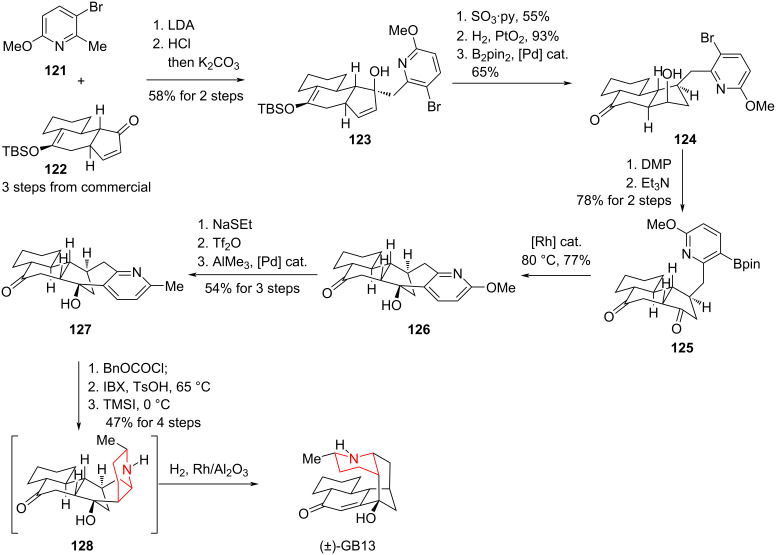
Total synthesis of GB13 by Sarpong and co-workers.

In 2009, Sarpong and co-workers reported a total synthesis of the galbulimima alkaloid GB13. Mander and co-workers had already completed an earlier total synthesis of GB13 in 2003 [86]. However, a key difference between the Sarpong and Mander strategies lies in the construction of the piperidine ring: Sarpong’s route features a catalytic hydrogenation of a pyridine precursor, whereas Mander’s strategy relied on an Eschenmoser fragmentation as well as a reductive amination (Scheme 17) [87].

Pyridine analogue **121** was converted to tetracyclic compound **123** via a 1,2-addition. A five-step transformation, including allylic hydroxy group transposition, olefin hydrogenation, and DMP oxidation, provided diketone **125**. The two carbonyl groups in **125** were then chemoselectively and stereoselectively reduced using rhodium as a catalyst to yield hydroxylated ketone **126**. A further three-step transformation provided the catalytic hydrogenation precursor **127**. Catalytic hydrogenation of **127** yielded the piperidine ring with a dr ratio of 8:1, completing the total synthesis of GB13 in overall 18 steps.

#### Total synthesis to the alkaloids GB13 by Shenvi, 2022

**Scheme 18:**
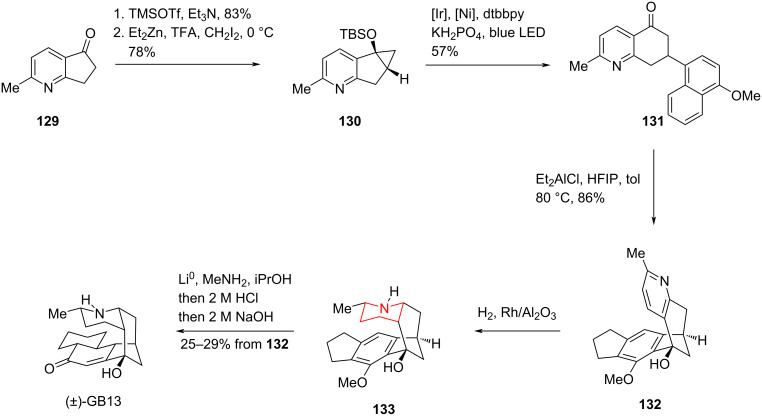
Total synthesis of GB13 by Shenvi and co-workers.

In 2022, Shenvi and co-workers completed another concise and efficient total synthesis of GB13 (Scheme 18) [88]. Starting from pyridine derivatives **129**, they obtained the key intermediate **131** via a Simmon–Smith reaction and a bimetallic-mediated photocatalytic radical coupling reaction. Intermediate **132** was then constructed by Friedel–Crafts alkylation using diethylaluminum chloride as a Lewis acid. The pyridine ring was then hydrogenated to the piperidine by direct hydrogenation, and the benzene ring was then reduced to an unsaturated ketone structure by Birch reduction, completing the highly efficient total synthesis of GB13 in 6 steps with an overall yield of 10%.

#### Total synthesis of (±)-corynoxine and (±)-corynoxine B by Xia, 2014

**Scheme 19:**
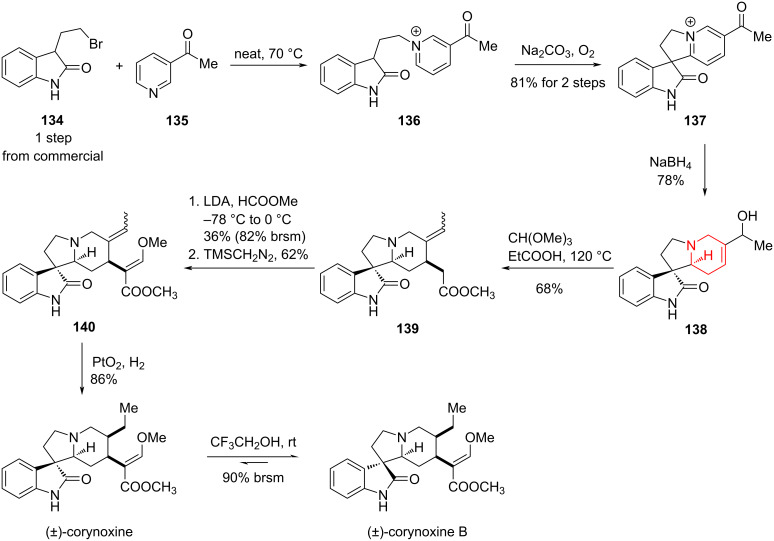
Total synthesis of (±)-corynoxine and (±)-corynoxine B by Xia and co-workers.

In 2014, Xia and co-workers completed an outstanding total synthesis of (±)-croynoxine and (±)-croynoxine B, two oxindoline-type tetracyclic natural products [89]. Starting from 2-oxoindole derivative **134** and the 3-substituted pyridine **135**, the pyridinium salt **136** was synthesized, which perfomed as a key substrate for the aerobic oxidation. The formation of the tetracyclic 3-spirooxindole structure **137** was achieved through a transition-metal-free intramolecular cross-dehydrogenative coupling. With **137** in hand, a sequence of transformations including ketone reduction with NaBH_4_, Johnson–Claisen rearrangement, enol ester formation, and methylation afforded **140**. Finally, hydrogenation of **140** via PtO_2_/H_2_ generated the target molecule (±)-corynoxine, and under acidic conditions, (±)-corynoxine could be isomerized to (±)-corynoxine B (Scheme 19).

#### Total synthesis of (+)-serratezomine E, (±)-serralongamine A and (−)-huperzine N by Bonjoch, 2015 and 2016

**Scheme 20:**
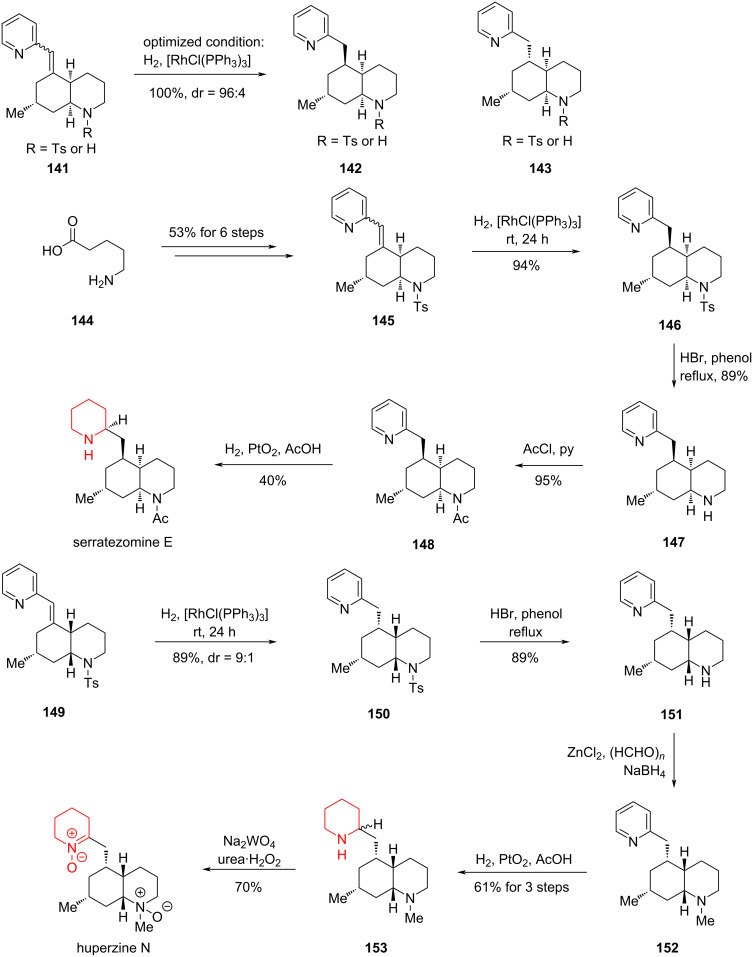
Total synthesis of (+)-serratezomine E and the putative structure of huperzine N by Bonjoch and co-workers.

In 2015, Bonjoch and co-workers reported a method for the selective hydrogenation of an exocyclic double bond conjugated to an aromatic ring [90]. For the pyridine derivative **141**, rhodium catalysis provided the chiral reduction product in quantitative yield and excellent stereoselectivity. Leveraging this unique reaction strategy, Bonjoch completed the total synthesis of the tricyclic natural product serratezomine E and the putative structure of huperzine N (Scheme 20).

Starting from simple 5-aminopentanoic acid (**144**), they synthesized the reduction precursor **145** in 53% yield over six steps. Subsequent catalytic hydrogenation afforded the diastereomeric product **146** in high yield. With compound **146** in hand, they completed the total synthesis of serratezomine E through a three-step sequence comprising removal of the Ts group, acetylation, and pyridine hydrogenation. Simultaneously, starting from **149**, they employed a similar route involving rhodium-catalyzed double-bond reduction, nitrogen methylation, pyridine reduction, and formation of the nitrogen oxide to give the putative structure of huperzine N.

**Scheme 21:**
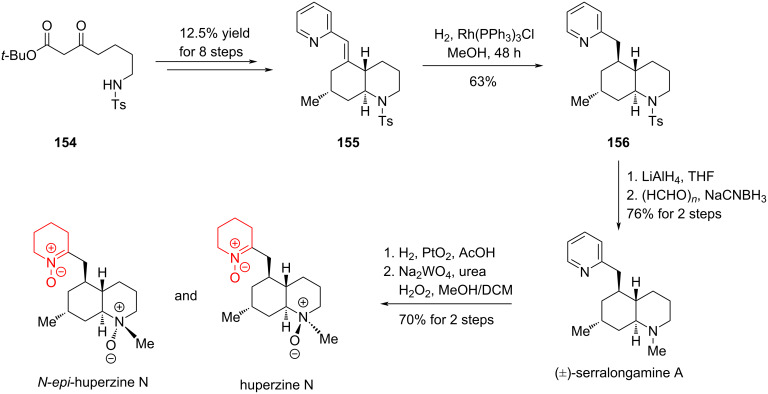
Total synthesis of (±)-serralongamine A and the revised structure of huperzine N and N-*epi*-huperzine N.

In 2016, Bonjoch and co-workers reported another synthesis work to revise the structure of huperzine N. Similar to their previous study, the synthesis started with a 1,3-dicarbonyl compound **154** to synthesize the hydrogenation precursor **155**. A three-step process, including hydrogenation of the olefin, hydride reduction and methylation, afforded (±)-serralongamine A. With (±)-serralongamine A in hand, huperzine N and N-*epi*-huperzine N could be obtained via a reduction–oxidation sequence [91] (Scheme 21).

#### Asymmetric synthesis towards indenopiperidine core of an 11-β-HSD-1 inhibitor, 2016

Growing interest has focused on inhibiting 11-β-hydroxysteroid dehydrogenase type 1 (11-β-HSD-1), a key enzyme that alleviates insulin resistance by lowering cortisol production. Compound **157** emerged as a promising 11-β-HSD-1 inhibitor candidate. In 2016, researchers from Boehringer Ingelheim Pharmaceuticals and the University of Pennsylvania reported a concise and stereoselective synthesis of **157**, which strategically combined a Pd-catalyzed pyridine C–H acylation and an Ir-catalyzed asymmetric hydrogenation of the aromatic core [92].

The synthesis began with the coupling of trisubstituted benzene **158** and 2-substituted pyridine **159**, furnishing the bicyclic intermediate **160**. Three subsequent steps (74% overall yield) then provided the key hydrogenation precursor **161**. Hydrogenation of **161** under heterogeneous catalytic conditions (Pd/C, Pt/C, Raney Ni, Rh/C, Ru/C) proved inefficient, giving complex mixtures that included partially or fully hydrogenated pyridines, over-reduced products and even dimers of reduced species.

**Scheme 22:**
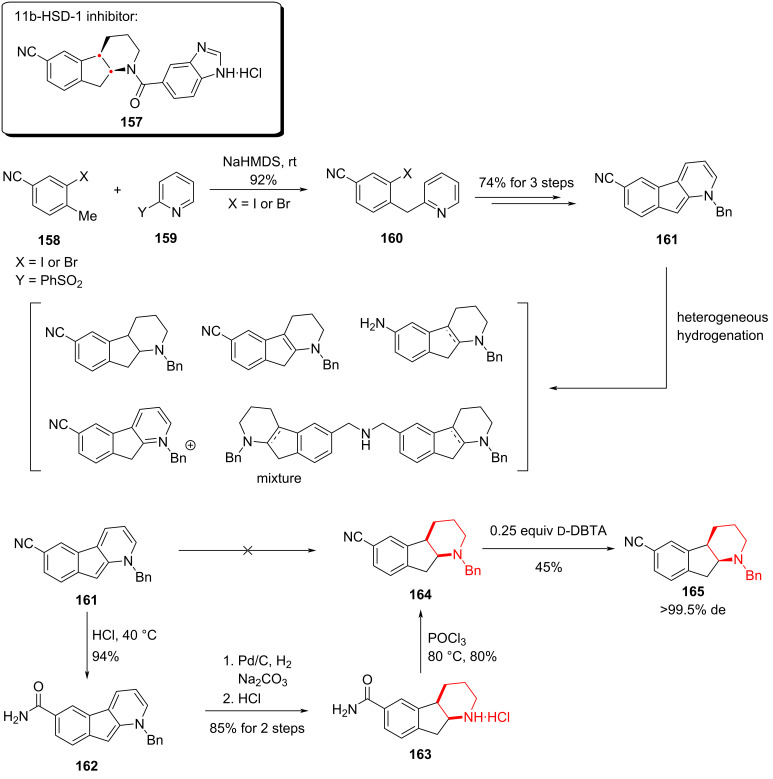
Early attempts to indenopiperidine core.

The authors proposed that the cyano substituent on the benzene ring might coordinate with or deactivate the metal catalysts, or undergo reduction under the reaction conditions, thereby diminishing catalytic activity. To address this challenge, they hydrolyzed the cyano group to an amide (**162**) under acidic conditions, which then allowed successful Pd/C-mediated hydrogenation to **163**. Subsequent dehydration with POCl_3_ and chiral resolution using ᴅ-DBTA provided the optically enriched compound **165** (Scheme 22).

**Scheme 23:**
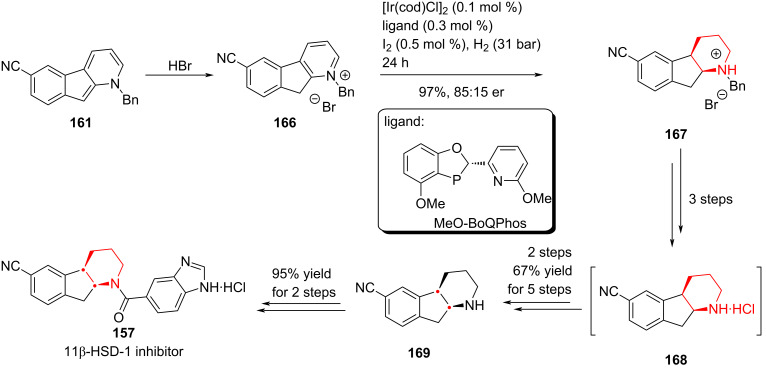
Homogeneous hydrogenation and completion of the synthesis.

While this approach rendered catalytic hydrogenation feasible, it also increased the complexity of the synthetic route, limiting its suitability for large-scale preparation of **157**. Consequently, the authors redirected their efforts toward homogeneous catalytic hydrogenation, a field that has seen rapid progress in recent years. Upon converting **161** into the zwitterionic salt **166**, extensive screening revealed that iridium catalysis with MeO-BoQPhos afforded the highest stereoselectivity. This approach furnished the reduced product **167** in high yield. After five further transformations (overall yield: 67%), the final coupling with a benzimidazole fragment efficiently delivered the target compound **157** (Scheme 23).

This study highlights the pivotal role of controlled aromatic hydrogenation, especially of pyridine derivatives, in modern drug discovery, and illustrates how the choice of the catalytic system (heterogeneous vs homogeneous) together with functional group management can decisively shape the success of complex molecule synthesis.

#### Total synthesis of jorunnamycin A and jorumycin by Stoltz, 2019

**Scheme 24:**
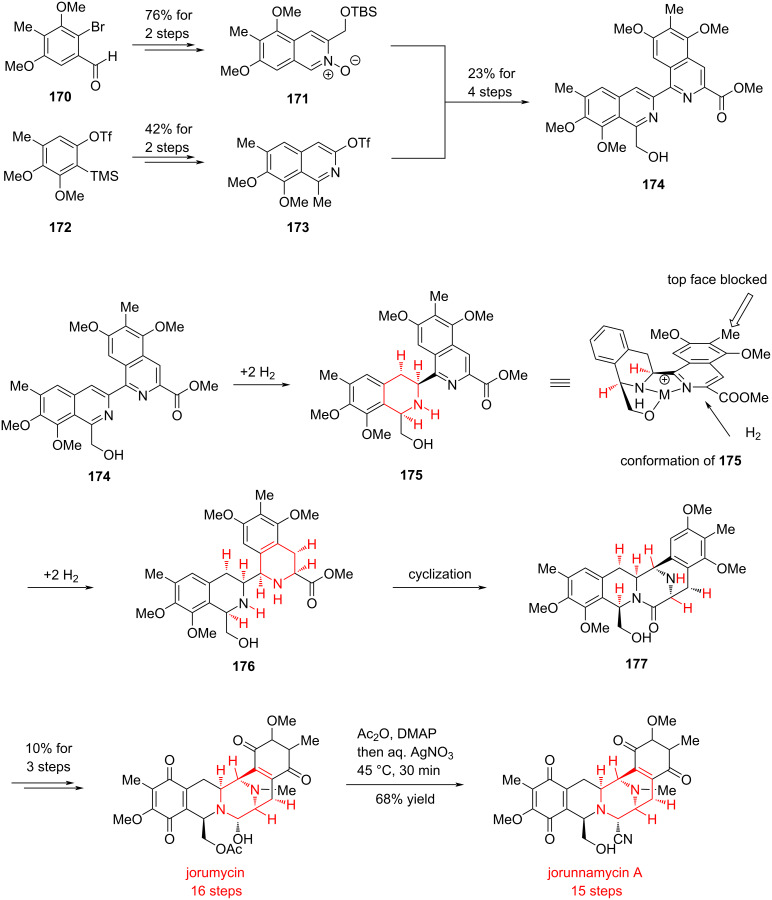
Total synthesis of jorunnamycin A and jorumycin by Stoltz and co-workers.

The bistetrahydroisoquinoline (bis-THIQ) alkaloids, a class of polycyclic natural products with significant physiological activities, have drawn sustained interest from synthetic chemists worldwide due to their pronounced antibacterial and anticancer properties. Since their discovery, bis-THIQ scaffolds have been constructed primarily through the Pictet–Spengler reaction, a cyclization strategy that continues to be widely employed in total synthesis. In 2019, Stoltz and co-workers developed an elegant substrate-directed asymmetric hydrogenation approach to construct the bis-THIQ framework, achieving the concise total syntheses of two structurally complex natural products [93] (Scheme 24).

Starting from the polysubstituted aromatic precursors **170** and **172**, the Stoltz group accomplished the synthesis of *N*-oxide **171** and isoquinoline compound **173** in two steps with 76% and 42% yield, respectively. These intermediates were then elaborated through a four-step sequence to afford the coupled intermediate **174** in 23% overall yield, which served as the substrate for catalytic hydrogenation.

During the hydrogenation of compound **174**, Stoltz and co-workers observed that the addition of the first two molecules of hydrogen to the substrate proceeded with high stereoselectivity. They proposed that this selectivity arises from chelation between the nitrogen and oxygen atoms from the substrate and the metal catalyst, which fixed the conformation of the polycyclic scaffold. As a result, the top face is sterically less favored, thereby hydrogen approaches from the opposite face. The subsequent addition of another two equivalents of hydrogen also supported this mechanistic hypothesis. Based on this stereochemical control, the Stoltz team successfully obtained the asymmetric hydrogenation product **176**, which spontaneously underwent intramolecular cyclization to furnish the bridged cyclic compound **177**.

With compound **177** in hand, a series of three transformations, including hydroxylation of aryl halide, partial lactam reduction with cyanide trapping, and oxidation of the phenol, enabled the total synthesis of jorunnamycin A in 15 steps. The acetylation of the hydroxy group in jorunnamycin A followed by cyano hydrolysis led to the total synthesis of another natural product, jorumycin.

#### Total synthesis of (−)-finerenone by Aggarwal, 2021

(−)-Finerenone is a non-steroidal mineralocorticoid receptor antagonist currently under investigation for the treatment of chronic kidney disease (CKD) associated with type 2 diabetes. Its molecular structure features a rare dihydronaphthyridine core, which presents a unique synthetic challenge. Given that (−)-finerenone is presently undergoing phase III clinical trials, the development of efficient and scalable methods for constructing the dihydronaphthyridine scaffold has become a focal point of interest in synthetic chemistry. In 2020, Aggarwal and co-workers reported a concise and enantioselective synthesis of (−)-finerenone via an asymmetric hydrogen atom transfer (HAT) strategy, completing the total synthesis in just six steps with high efficiency and stereoselectivity [94].

**Scheme 25:**
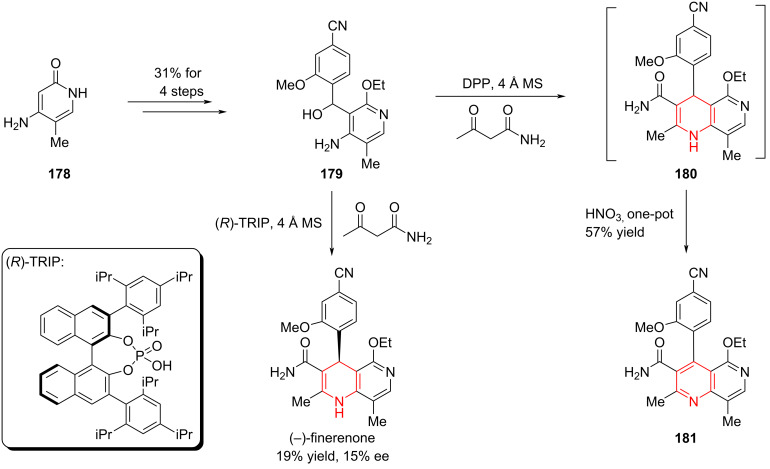
Early attempt towards (−)-finerenone by Aggarwal and co-workers.

Starting from the 2-pyridone derivative **178**, the authors synthesized the bicyclic intermediate **179** in four steps with an overall yield of 31%. Initially, they attempted a formal [4 + 2] cycloaddition between **179** and a 1,3-dicarbonyl compound, catalyzed by the chiral phosphoric acid (*R*)-TRIP, aiming to construct (−)-finerenone in a single step. However, this approach delivered the target compound in only 19% yield and a low 15% ee (Scheme 25). Given that high enantiopurity is critical for subsequent clinical studies, the authors needed to revise their strategy.

Instead of pursuing a direct asymmetric cyclization, they performed a formal [4 + 2] cycloaddition between **179** and the dicarbonyl compound, followed by in-situ oxidation of the resulting intermediate **180** to afford compound **181** in a one-pot sequence. At this step, the key challenge was to obtain (−)-finerenone with high enantioselectivity through asymmetric hydrogenation of intermediate **181** (Scheme 25).

During attempts to reduce intermediate **181** via asymmetric hydrogen atom transfer (HAT), the authors found that **181** exists as a racemic mixture, with chirality originating from its axially chiral biaryl structure. Owing to the steric hindrance introduced by substituents, **181** exists as a pair of atropisomers, which inspired a new strategy for achieving asymmetric hydrogenation.

The authors proposed that the two atropisomers exhibit different reactivity and selectivity under catalytic conditions, enabling a process of kinetic resolution. More importantly, they envisioned that this inherent resolution could be transformed into a dynamic kinetic resolution, thereby allowing for the selective formation of optically enriched finerenone.

**Scheme 26:**
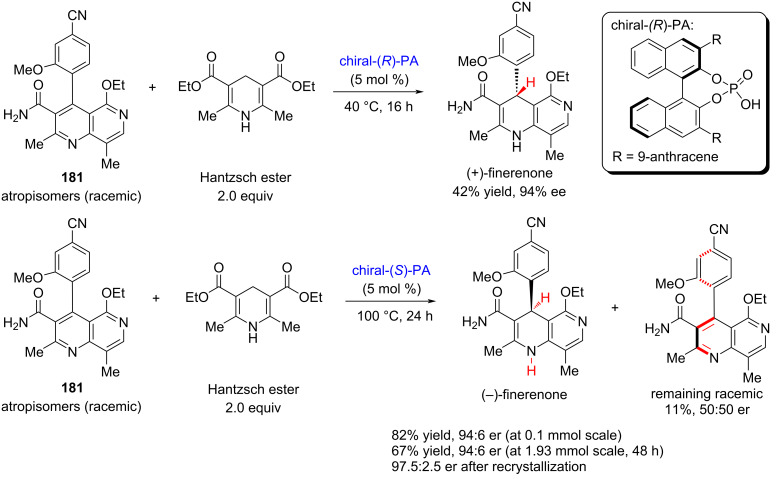
Enantioselective synthesis towards (−)-finerenone.

Subsequent experiments confirmed this hypothesis: by employing chiral phosphoric acids of different configurations as catalysts, the authors successfully obtained (+)-finerenone in 42% yield with 94% ee, and (−)-finerenone in 67–82% yield with 94:6 er, respectively (Scheme 26).

#### Total synthesis of (+)-*N*-methylaspidospermidine by Smith and Grigolo, 2022

**Scheme 27:**
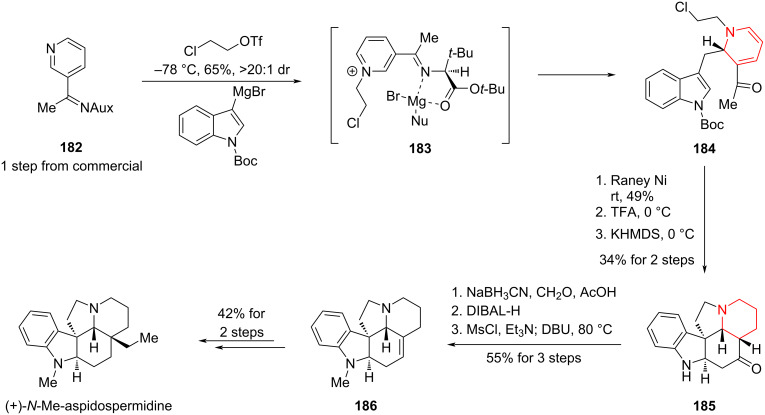
Total synthesis of (+)-*N*-methylaspidospermidine by Smith, Grigolo and co-workers.

In 2021 and 2022, Smith, Grigolo and co-workers reported a total synthesis of the monoterpene indole alkaloid *N*-methylaspidospermidine (Scheme 27) [95–96].

In a single-step reaction, pyridine **182** was activated with 2-chloroethyl triflate and the resulting pyridinium salt was dearomatized with a Grignard reagent to produce ketone **184**. In this step, the Grignard nucleophile added regio- and diastereoselectively at the 2-position of the pyridinium, consistent with established reactivity models from prior studies [97]. A three-step sequence involved Raney Ni hydrogenation of the dihydropyridine, TFA-mediated indole deprotection, and base-promoted formation of a C3 quaternary carbon center provided piperidine compound **185**. The resulting intermediate was trapped with a side chain enolate (derived from a methyl ketone), successfully constructing the pentacyclic aspidospermidine core. The next three steps comprised nitrogen methylation, ketone reduction, and hydroxyl elimination to afford **186**. In the final two steps, an ethyl group was introduced via Fe-mediated HAT and sulfone cleavage. This 10-step asymmetric synthesis demonstrates that, in the case of indole monoterpene alkaloids, the rational application of aromatic ring hydrogenation can markedly reduce step count and enhance overall efficiency.

#### Total synthesis of matrine-type alkaloids by Reisman, 2022

The tetracyclic alkaloids (+)-matrine and (+)-isomatrine, isolated from *Sophora flavescens*, are thought to originate biosynthetically from (−)-lysine. In 2022, Reisman and co-workers reported a pyridine hydrogenation strategy that provided collective access to matrine-type alkaloids [98]. That same year, Sherburn and co-workers described a complementary synthesis employing an aza-Diels–Alder cycloaddition followed by olefin hydrogenation [99].

**Scheme 28:**
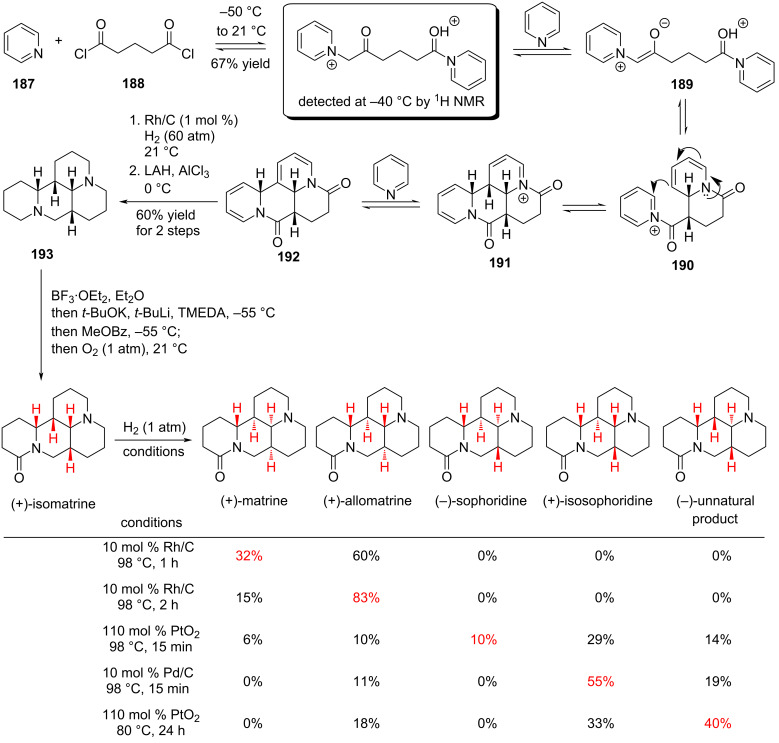
Dearomatization approach towards matrine-type alkaloids.

Starting from pyridine and glutaryl chloride, tetracyclic intermediate **192** was efficiently generated in a single step through sequential nucleophilic attack and intramolecular cyclization. (+)-Isomatrine was subsequently synthesized through catalytic hydrogenation via Rh/C, LAH reduction, and tertiary amine oxidation (Scheme 28).

After synthesizing (+)-isomatrine, the effects of catalyst type, loading, temperature, and reaction time on the hydrogenation process were systematically examined. The results revealed that appropriate tuning of the conditions enabled the selective synthesis of different natural products, including (+)-matrine, (+)-allomatrine, and other tetracyclic alkaloids, thereby achieving an efficient collective synthesis of matrine-type alkaloids.

#### Asymmetric total synthesis of senepodine F by Ishikawa, 2023

**Scheme 29:**
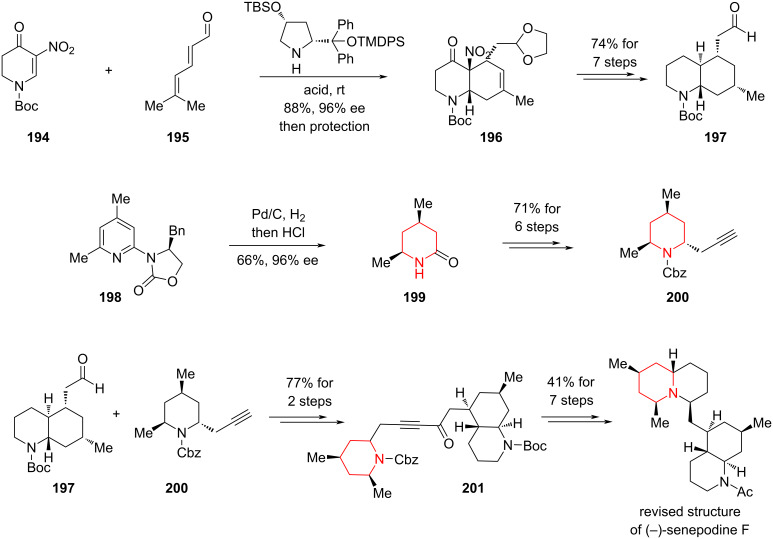
Asymmetric total synthesis to (−)-senepodine F via an asymmetric hydrogenation of pyridine.

*Lycopodium* alkaloids, isolated from plants of the *Lycopodium* genus (*Lycopodiaceae* family), represent a vast and structurally diverse family of natural products. To date, over 500 members of this family have been reported, with some known to exhibit acetylcholinesterase inhibitory activity and other notable bioactivities. Their architecturally complex and unique polycyclic frameworks have attracted sustained interest from synthetic, natural product, and medicinal chemists alike. In 2023, Ishikawa and co-workers reported the first asymmetric total synthesis of (−)-senepodine F, a tetracyclic *Lycopodium* alkaloid (Scheme 29) [100].

The synthesis commenced with a stereoselective Diels–Alder reaction between compounds **194** and **195** under asymmetric amine catalysis, directly affording the bicyclic intermediate **196**, which was further elaborated over seven steps (overall yield: 74%) to give **197**. In parallel, the authors adopted Glorius’ protocol, applying an Evans chiral auxiliary to achieve asymmetric catalytic hydrogenation of the pyridine derivative **198**, delivering the lactam **199** in 66% yield and with excellent enantioselectivity (96% ee). Subsequent transformations over six steps (overall yield: 71%) provided the piperidine building block **200**.

Coupling of the key fragments **197** and **200** was achieved in two steps (77% yield), affording alkyne intermediate **201**. This was followed by a sequence of strategic functional group manipulations, including a pivotal intramolecular S_N_2 cyclization, ultimately completing the total synthesis of (−)-senepodine F. Notably, this work also led to the structural revision of the originally assigned natural product, as reported in earlier isolation studies.

#### Hydrogenation strategy in total synthesis by Qi, 2023 and 2024

Indole-containing natural products have long attracted considerable interest in synthetic chemistry, owing to their pronounced biological activities and the biosynthetic relevance of indole as a key metabolite in diverse pathways. These biosynthetic processes frequently inspire new synthetic methodologies and retrosynthetic strategies. Simple indole derivatives – such as indole-3-acetic acid, tryptophan, and indole-2-carboxylic acid – are accessible on a large scale via biological routes. Nevertheless, for arenes containing both a benzene ring and a heteroaromatic core, achieving regioselective catalytic hydrogenation that discriminates between the phenyl and heteroaromatic moieties remains a persistent challenge.

**Scheme 30:**
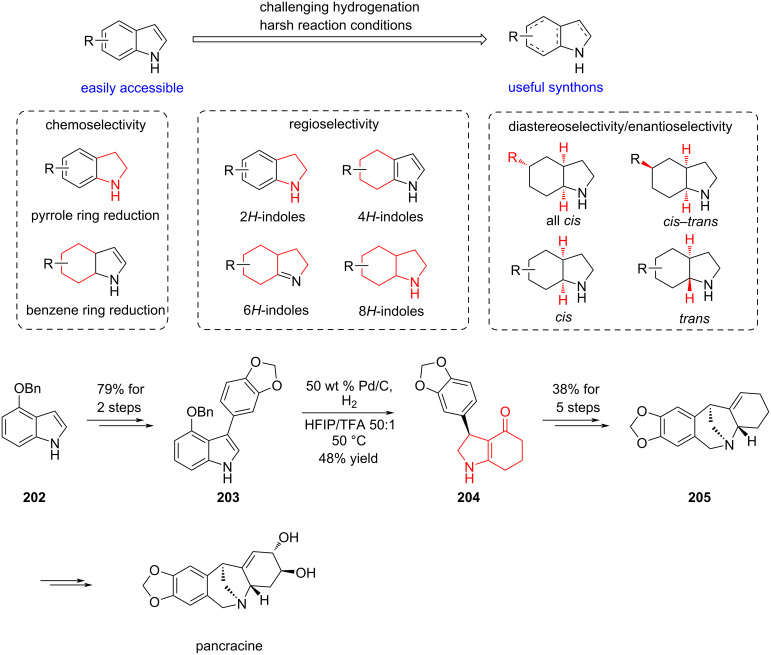
Selective hydrogenation of indole derivatives and application.

A recent work by Qi and co-workers has substantially advanced the selective hydrogenation of indole-containing compounds. Systematic tuning of reaction parameters such as catalyst loading, solvent, reaction time, and temperature enabled the selective access to diverse hydrogenated products. These developments have established a valuable foundation for the total synthesis of indole-derived alkaloids, facilitating more concise and efficient synthetic routes (Scheme 30) [101].

As an illustration, compound **203** underwent hydrogenation with 50 wt % Pd/C in HFIP/THF 50:1 to give the debenzyloxylated product **204** in 48% yield. The reaction selectively reduced the indole ring while preserving the conjugated olefin adjacent to the carbonyl group, thereby providing a valuable synthetic handle that was exploited in the formal synthesis of pancracine.

**Scheme 31:**
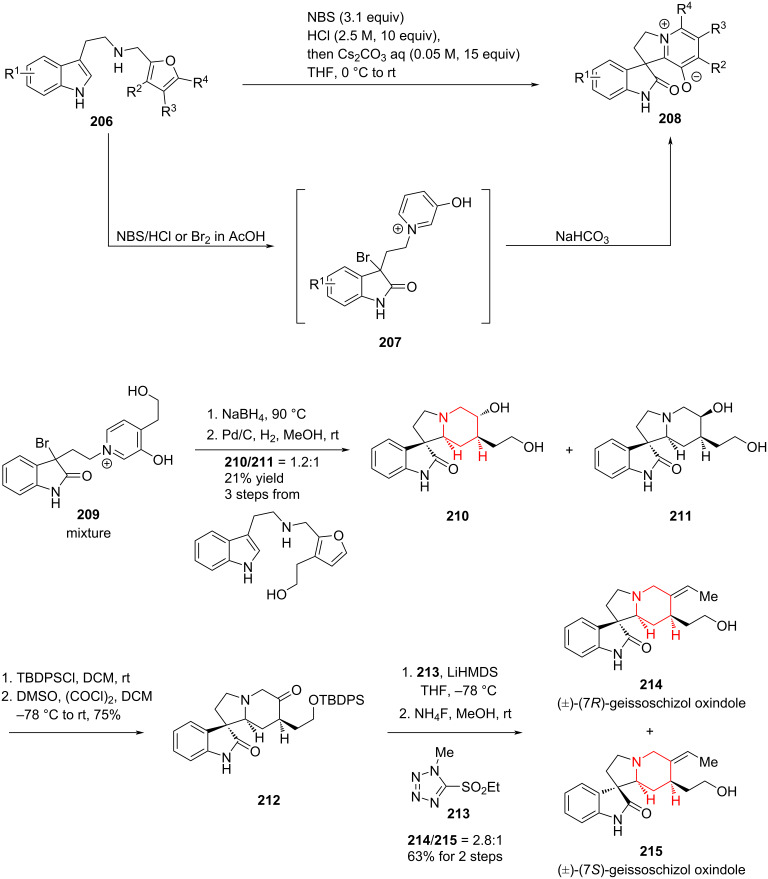
Synthetic approaches to the oxindole alkaloids by Qi and co-workers.

Skeletal rearrangements enable the rapid conversion of simple molecules into complex architectures and represent a powerful strategy in natural product synthesis. Qi and co-workers recently reported a one-pot reaction featuring a double oxidative rearrangement cascade of furans and indoles followed by nucleophilic cyclization. This methodology was applied to the formal synthesis of rhynchophylline/isorhynchophylline and the first total syntheses of (±)-(7*R*)- and (±)-(7*S*)-geissoschizol oxindoles (Scheme 31) [102].

In the presence of NBS as the oxidant, precursor **206** undergoes an aza-Achmatowicz rearrangement to give the monobrominated intermediate **207**, which upon treatment with NaHCO_3_ furnishes the spirocyclic scaffold **208**. From this versatile intermediate, a range of oxindole natural products can be accessed. Notably, compound **208** also can be selectively hydrogenated either at the pyridine or indole ring by adjusting the reaction conditions, thereby enabling divergent access to a range of related natural products.

#### Total synthesis of annotinolide B via sequential quinoline dearomatization, Smith, 2025

**Scheme 32:**
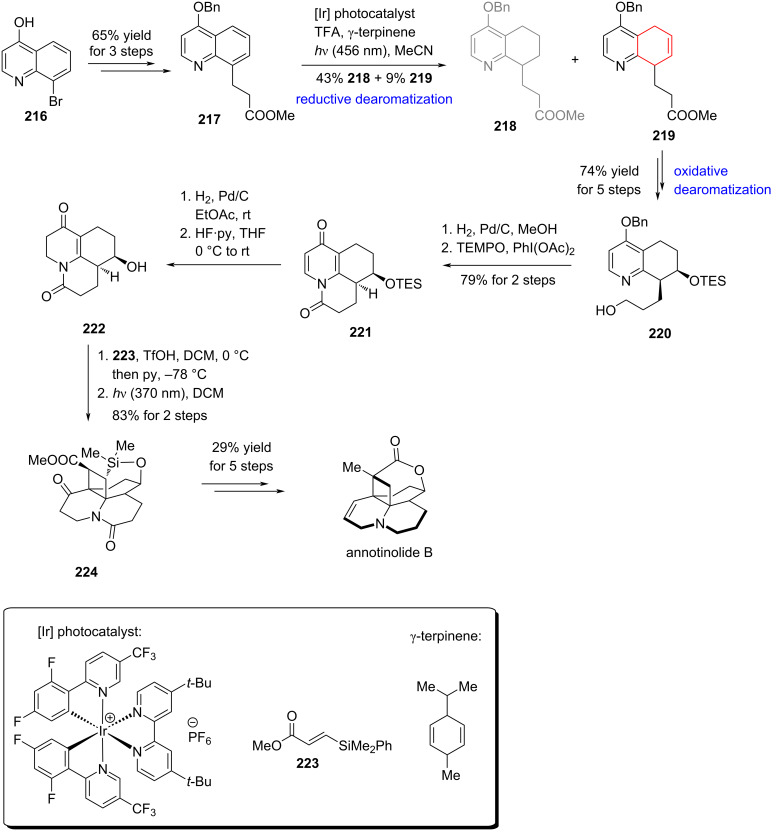
Total synthesis of annotinolide B by Smith and co-workers.

Very recently, Smith and co-workers disclosed an elegant total synthesis of the cyclobutane-containing Lycopodium alkaloid annotinolide B (Scheme 32) [103]. Beginning from bromo-substituted quinoline **216**, the authors prepared methyl ester **217** through a concise three-step sequence. A key photochemical dearomatization – originally developed by Ma and co-workers in 2023 – then transformed **217** into dihydroquinoline **219**, accompanied by a minor amount of tetrahydroquinoline **218** [104]. Subsequent transformations over five steps delivered protected intermediate **220** with a primary alcohol group, which was debenzylated and the resulting pyridone intermediate subjected to oxidative cyclization to provide tricyclic intermediate **221**. From this scaffold, a sequence comprising olefin hydrogenation, a [2 + 2] cycloaddition, Pd-catalyzed reduction, Vaska’s catalyst-mediated amide reduction, and final lactonization furnished annotinolide B in 20 steps and 2.7% overall yield.

## Conclusion

Over the past decade, advances in catalyst platforms and ligand architecture have transformed arene hydrogenation from a niche reactivity into a broadly general strategy. Enantioselective hydrogenations now encompass quinolines, isoquinolines, benzofurans, pyridines, and related heteroarenes, with stereocontrol enabled by finely tuned ligand classes – N-heterocyclic carbenes, chiral diamines, bisoxazolines, bisphosphines, and pincer frameworks. These systems create discriminating steric/electronic environments and compatible H_2_-activation manifolds that deliver site-, chemo-, and enantioselectivity even in the presence of coordinating heteroatoms.

Critically, these developments have moved beyond method demonstration to strategic application in complex settings. Arene hydrogenation is increasingly used to install multiple stereocenters in a single operation, streamline protecting-group and redox economies, and unlock divergent routes from common intermediates – thereby improving step economy and scalability in total synthesis and medicinal chemistry. For example, Stoltz and co-workers leveraged a substrate-directed asymmetric hydrogenation of isoquinolines to accomplish concise total syntheses of jorunnamycin A and jorumycin, providing a compelling alternative to the Pictet–Spengler reaction and underscoring the strategic value of arene hydrogenation in modern synthesis.

Despite the impressive advances achieved thus far, several fundamental questions remain unresolved and continue to shape the research frontier. A critical issue concerns the influence of substituents with distinct electronic and steric properties on hydrogenation reactivity and selectivity; subtle differences in substitution patterns can dramatically alter reaction pathways, yet predictive models remain underdeveloped. Equally important is the role of heteroatoms, whose tendency to coordinate to transition-metal centers may attenuate catalytic activity or redirect selectivity. Even the archetypal aromatic substrate – benzene – poses a formidable challenge: achieving asymmetric hydrogenation under mild and practical conditions remains an unmet goal. Beyond these challenges, the broader problems of chemo-, regio-, and stereoselectivity underscore the need for new paradigms in catalyst design.

Looking ahead, future progress will hinge on three interrelated directions. First, the deployment of earth-abundant, inexpensive metals will be essential to reduce cost and ensure sustainability, thereby expanding the scope of practical applications. Second, innovation in catalyst and ligand architecture must aim not only at higher levels of stereo- and site-selectivity but also at expanding functional group tolerance and enabling transformations previously considered inaccessible. Third, deeper mechanistic insight into the interplay between substituents, electronic structure, and catalyst behavior is urgently required, as this knowledge will guide the rational design of more general catalytic systems.

With sustained methodological innovation, asymmetric hydrogenation of arenes is poised to evolve from a specialized transformation into a central strategy in synthesis. Its impact will likely extend far beyond methodological studies, driving progress in the streamlined construction of natural products, the design of bioactive molecules, and the discovery of new therapeutic agents. In this sense, the next decade holds the promise not merely of incremental improvements, but of a paradigm shift in how chemists harness arene hydrogenation in complex molecule synthesis.

## Data Availability

Data sharing is not applicable as no new data was generated or analyzed in this study.
